# Supplementary figures and images for: Constructive connectomics: How neuronal axons get from here to there using gene-expression maps derived from their family trees
Source: PLoS Comput Biol. 2022 Aug 25;18(8):e1010382. doi: 10.1371/journal.pcbi.1010382 (PMC9409546; doi:10.1371/journal.pcbi.1010382)

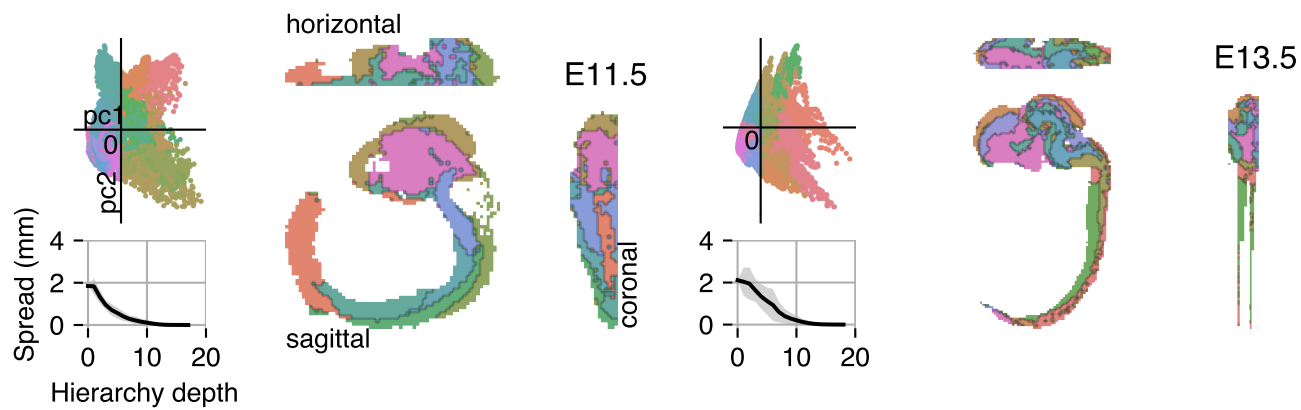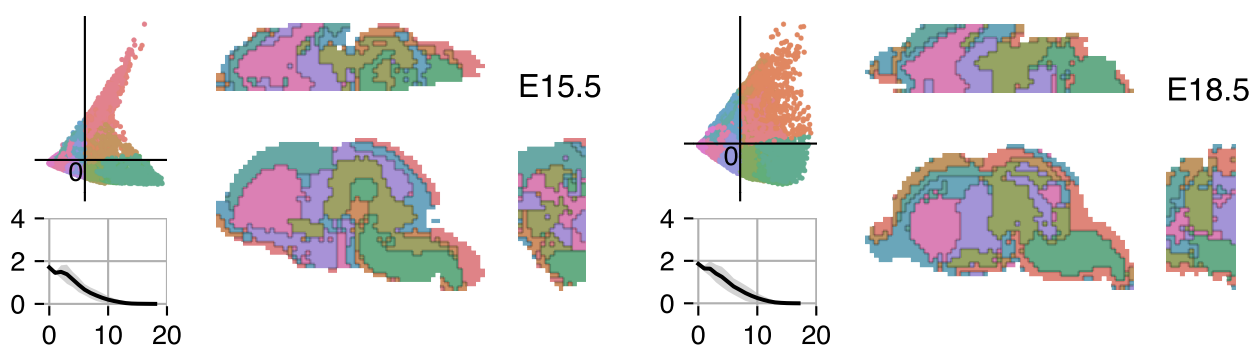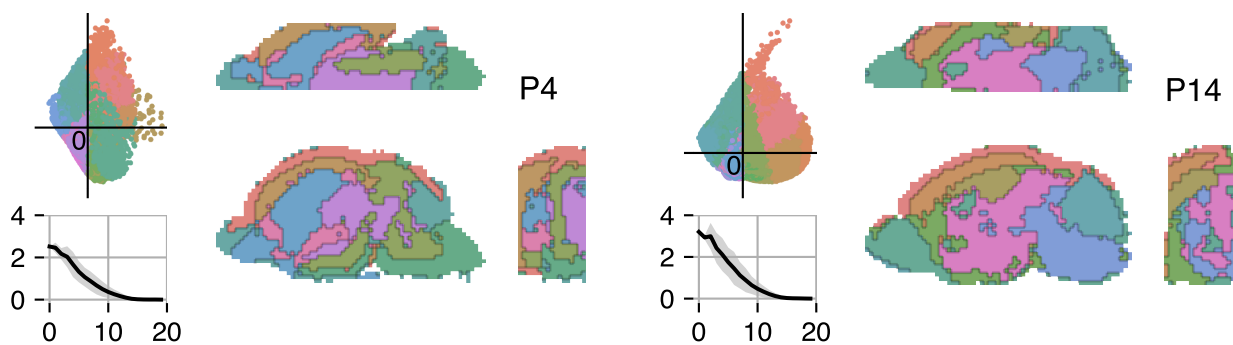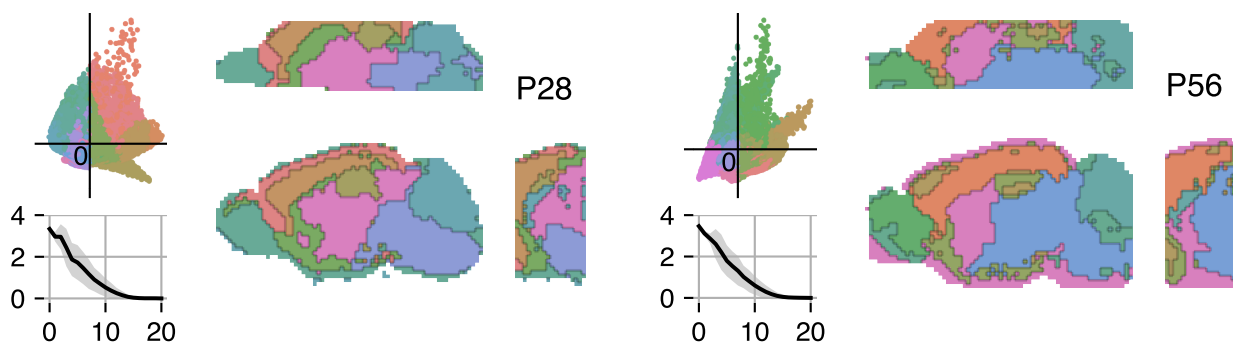

Supplement: S1 Fig — Only depth 3—the bottom tile in Fig 5—is shown, but other depths can be inferred by grouping similar colors. Decompositions were performed independently of one another (unlike Fig 6, where established hierarchies are projected across time points). The spatial spread of hierarchical regions goes down with hierarchy depth at each measured time point. (PDF) [file pcbi.1010382.s001.pdf]

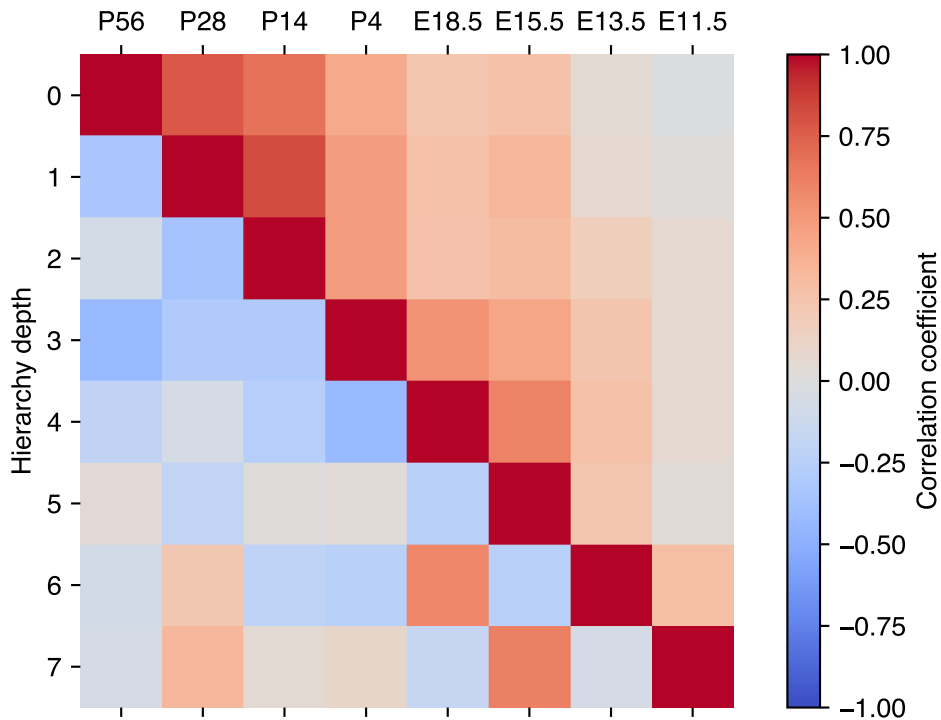

Supplement: S2 Fig — Upper triangle Pairwise correlation coefficient between the estimated asymmetries Cov→ measured at the root of the hierarchies at various time points. Although the asymmetry measurement is done independently at each time point, the main direction of covariance across all voxels is correlated. Generally, nearby time points are more correlated than distant time points. This correlation is surprising a priori, because the absolute gene expression changes from E11.5 to P56. Lower triangle Pairwise correlation coefficient between the estimated asymmetries Cov→ at the root of a hierarchy and other asymmetries within the same hierarchy. (Each column represents a time point, and each row a depth of the hierarchy, with the root at zero depth.) In contrast to standard principal component analysis, orthogonality between components is not enforced by our hierarchical decomposition. Nevertheless, we find that many pairs of components are poorly correlated. This implies that the direction of the strongest covariance is not along any single direction for all subsets of voxels, but is rotated in high-dimensional expression space at each iteration of the decomposition. The model assumes that differential gene expression vectors δ, and consequently the asymmetries Δ^ are independent. This matches the observation in the experimental data that the successive Cov→i are poorly correlated in expression space. The poor correlation is not by construction, because unlike PCA (Principal Component Analysis), orthogonality is not enforced by our decomposition. (PDF) [file pcbi.1010382.s002.pdf]

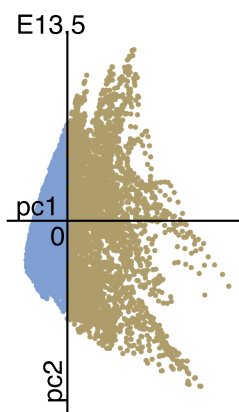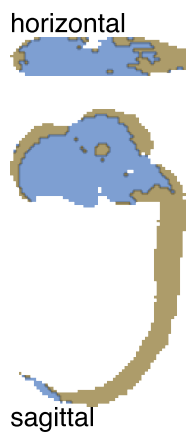

E13.5

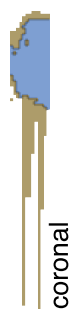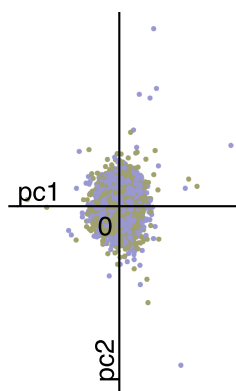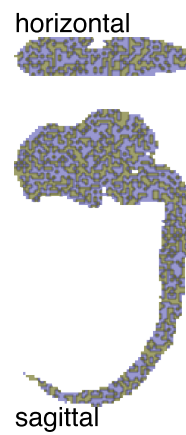

E13.5

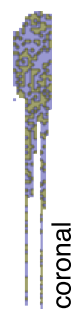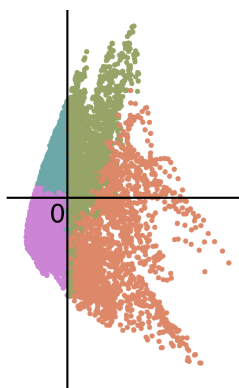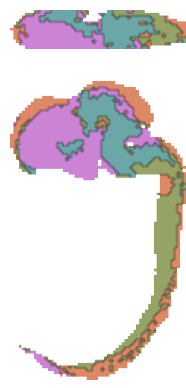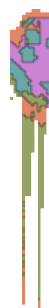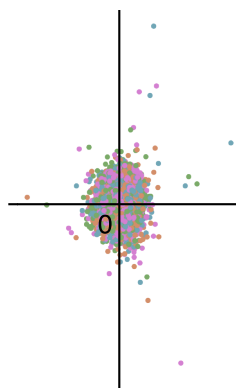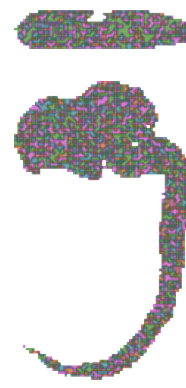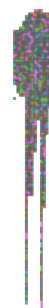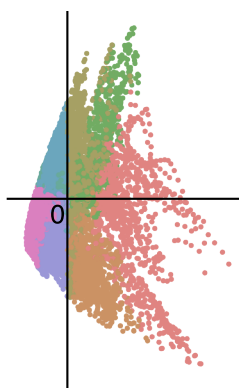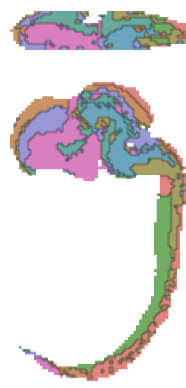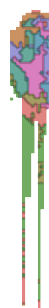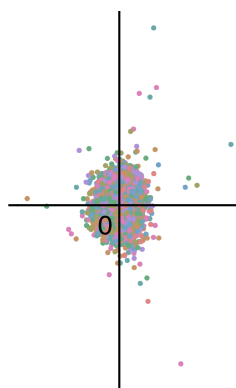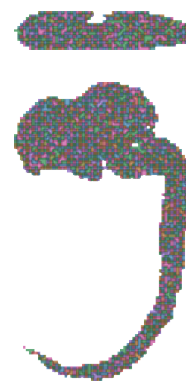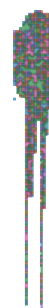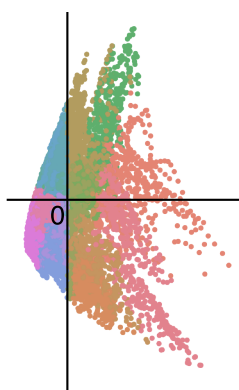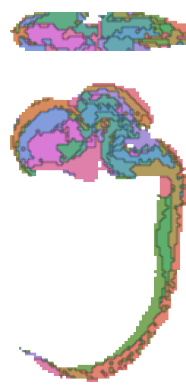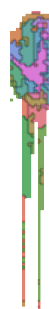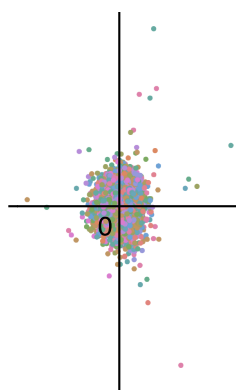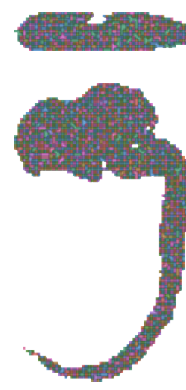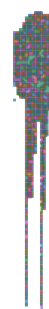

Supplement: S4 Fig — Analysis and depiction as in Fig 5. Bottom matrix shows pairwise correlation coefficient among components within the hierarchy at the displayed depths. (Similar to the bottom triangle in S2 Fig). (PDF) [file pcbi.1010382.s004.pdf]

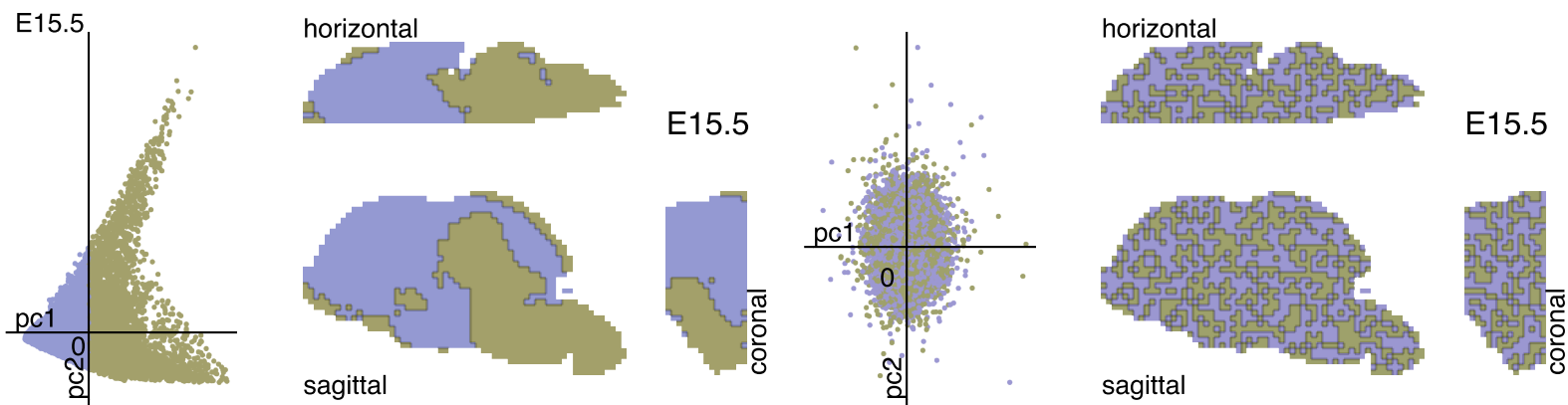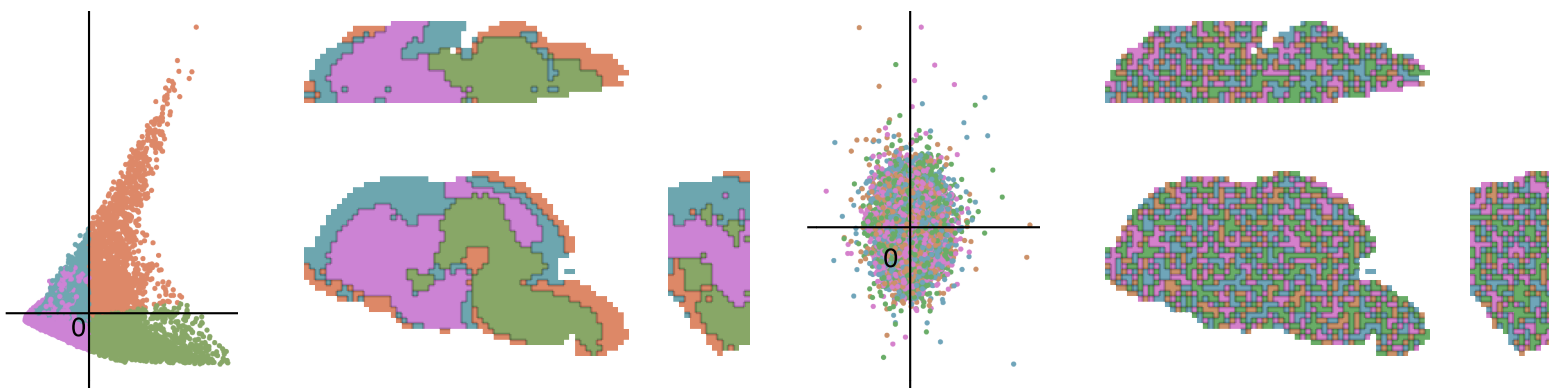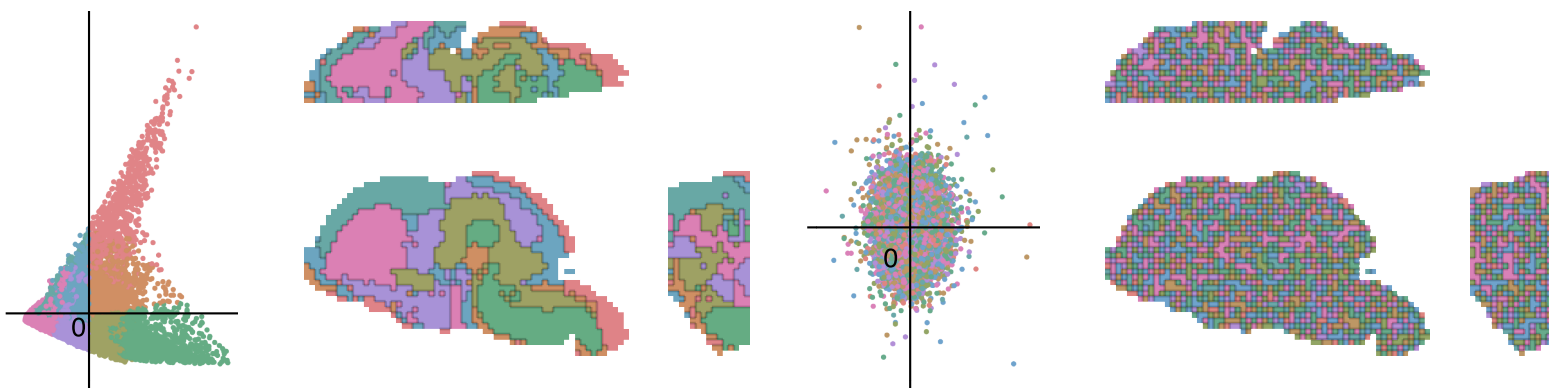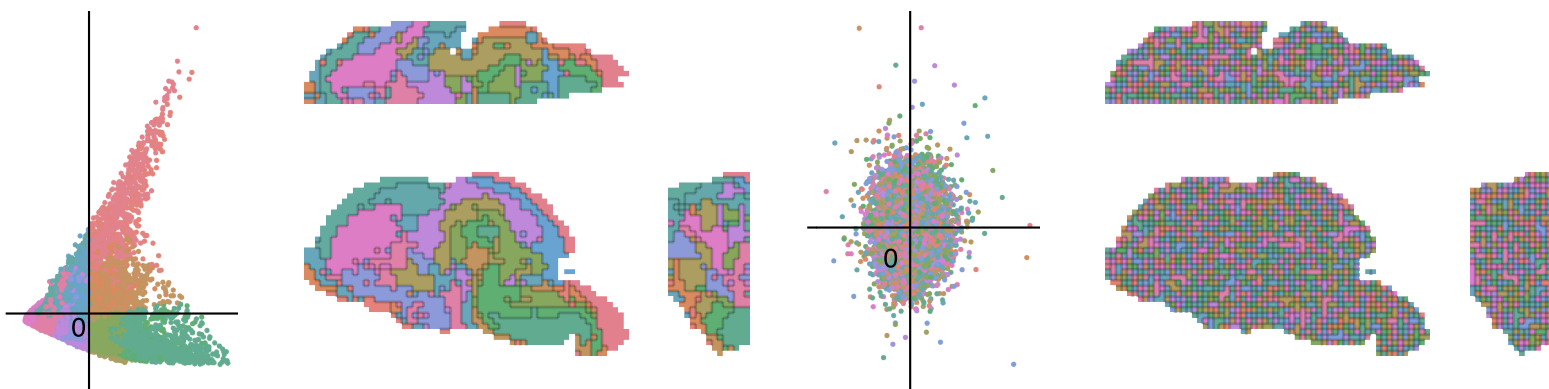

Supplement: S5 Fig — Analysis and depiction as in Fig 5. Bottom matrix shows pairwise correlation coefficient among components within the hierarchy at the displayed depths. (Similar to the bottom triangle in S2 Fig). (PDF) [file pcbi.1010382.s005.pdf]

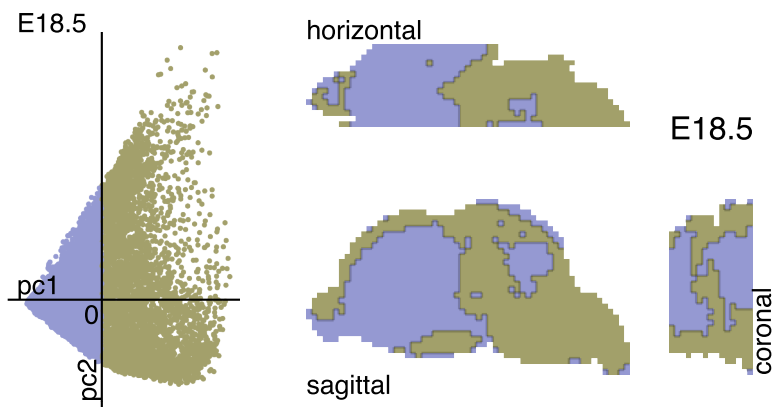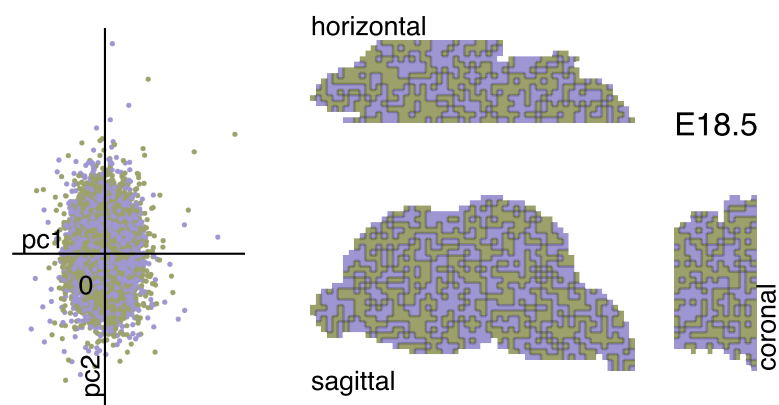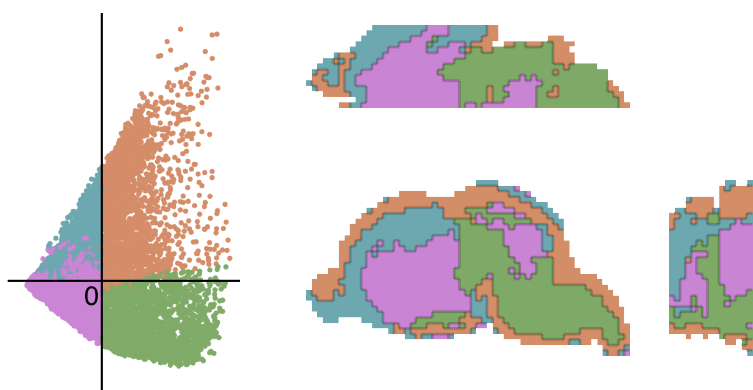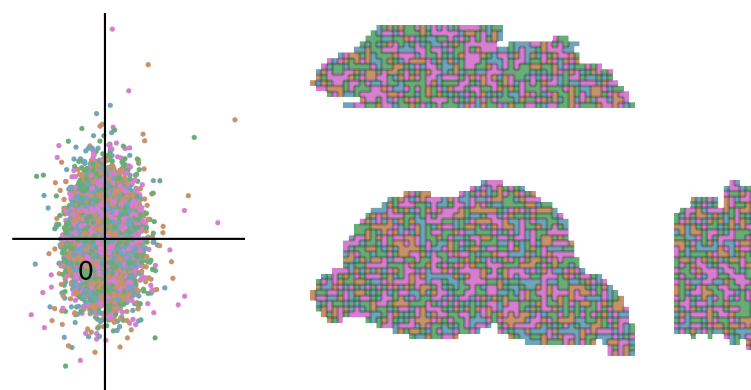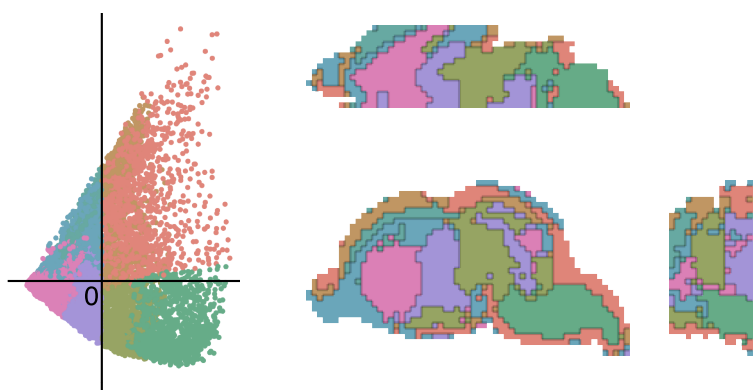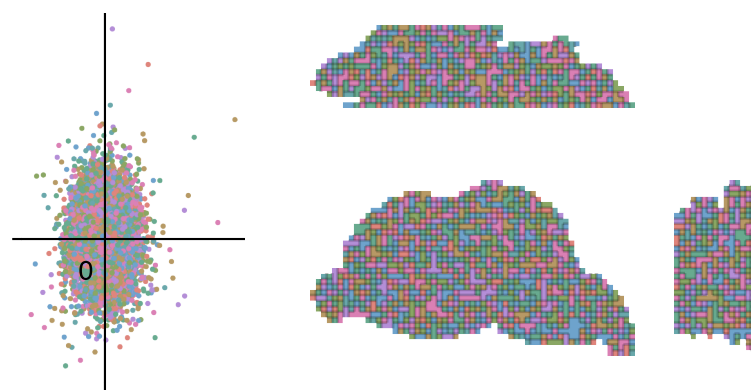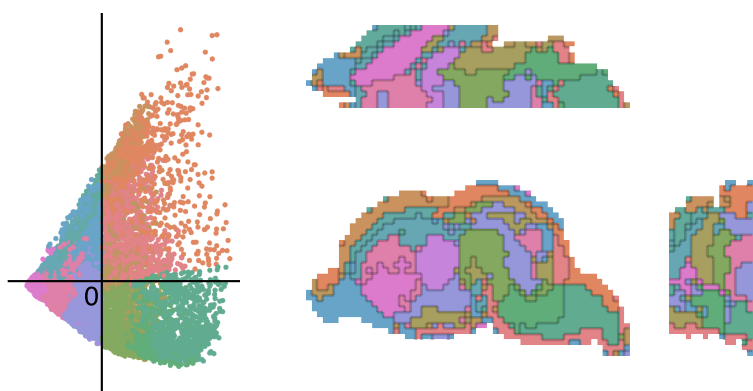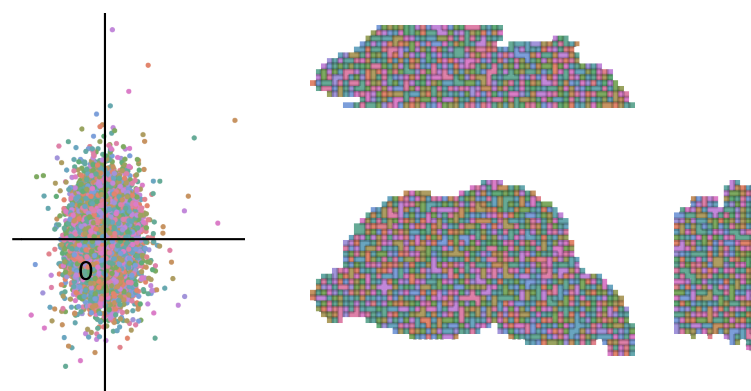

Supplement: S6 Fig — Analysis and depiction as in Fig 5. Bottom matrix shows pairwise correlation coefficient among components within the hierarchy at the displayed depths. (Similar to the bottom triangle in S2 Fig). (PDF) [file pcbi.1010382.s006.pdf]

P4

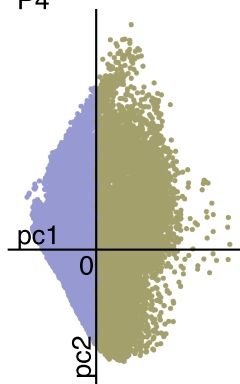

horizontal

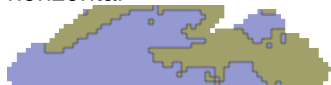

sagittal

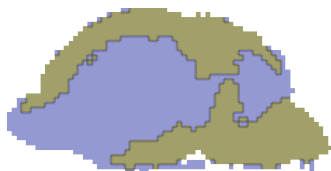

P4

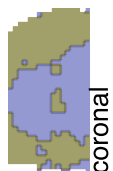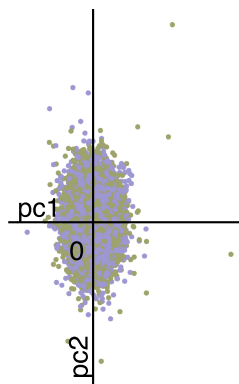

horizontal

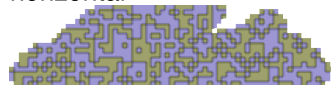

sagittal

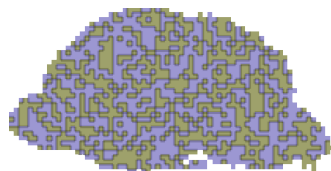

P4

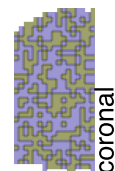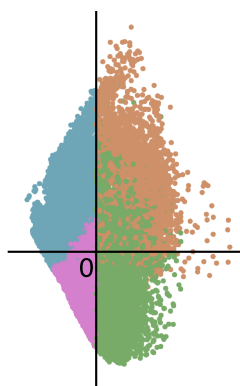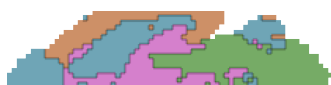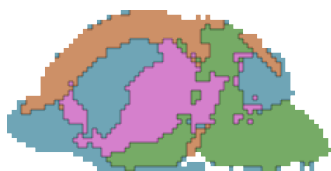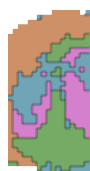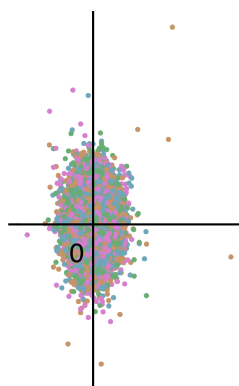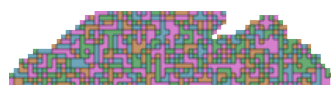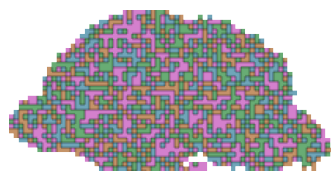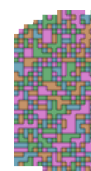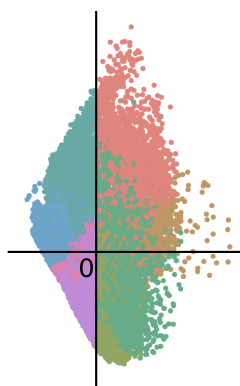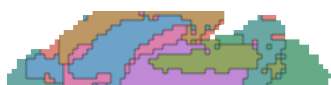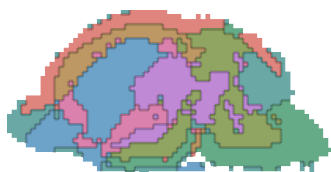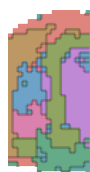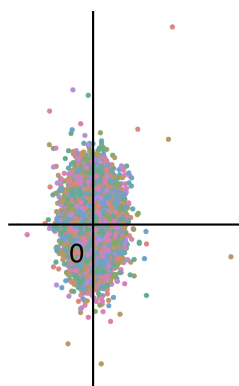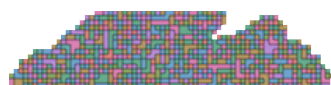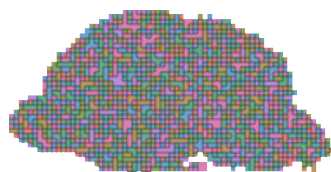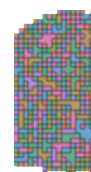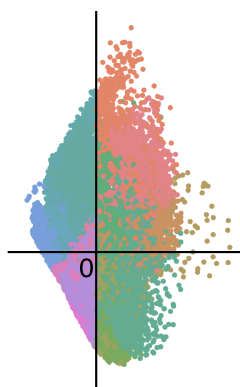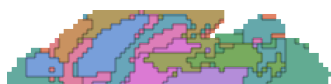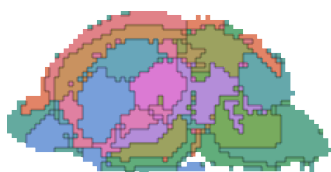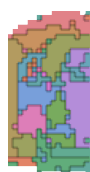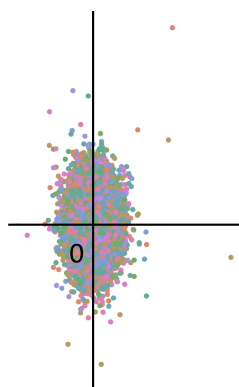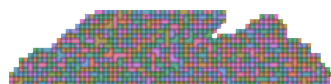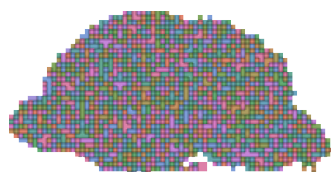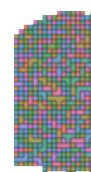

Supplement: S7 Fig — Analysis and depiction as in Fig 5. Bottom matrix shows pairwise correlation coefficient among components within the hierarchy at the displayed depths. (Similar to the bottom triangle in S2 Fig). (PDF) [file pcbi.1010382.s007.pdf]

P14

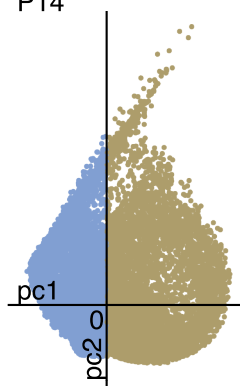

horizontal

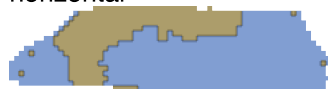

P14

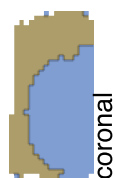

sagittal

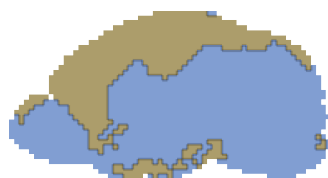

horizontal

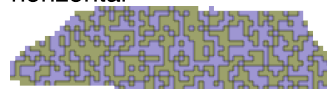

P14

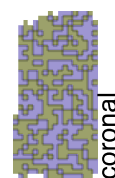

sagittal

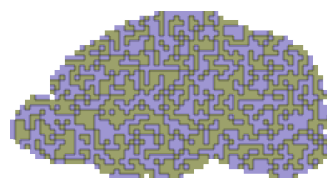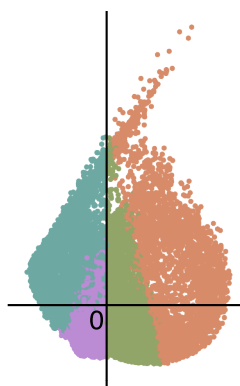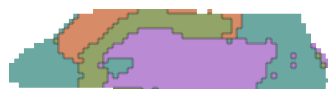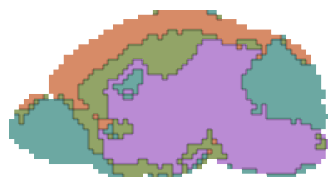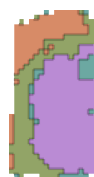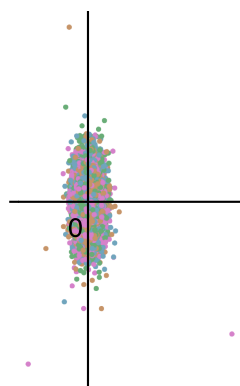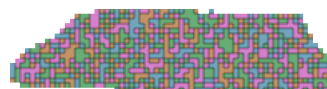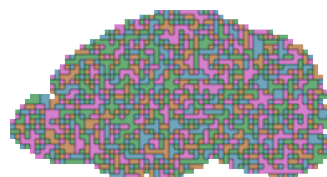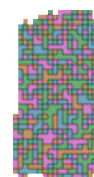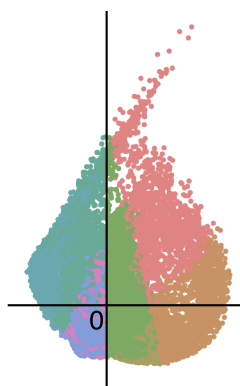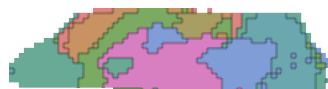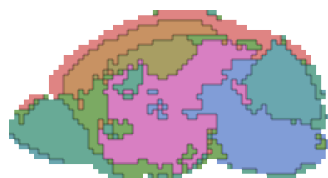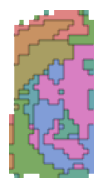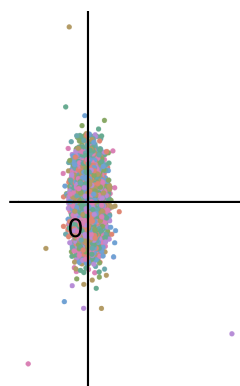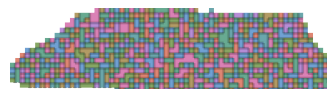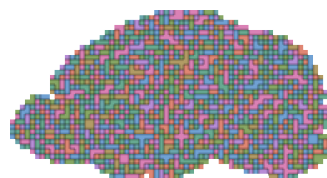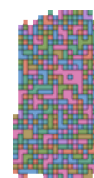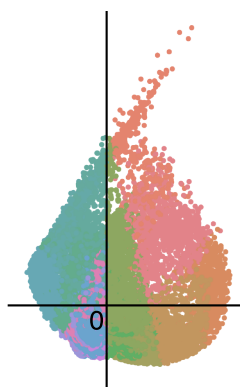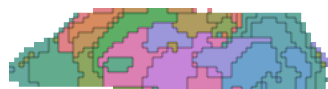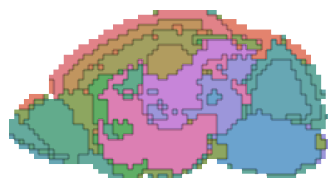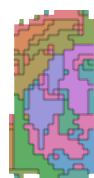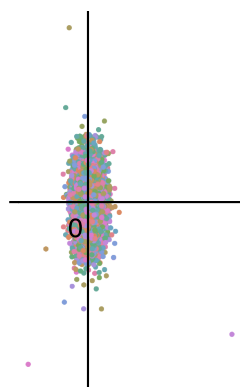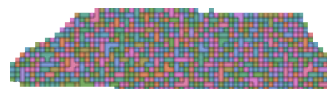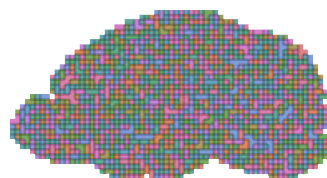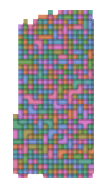

Supplement: S8 Fig — Analysis and depiction as in Fig 5. Bottom matrix shows pairwise correlation coefficient among components within the hierarchy at the displayed depths. (Similar to the bottom triangle in S2 Fig). (PDF) [file pcbi.1010382.s008.pdf]

P28

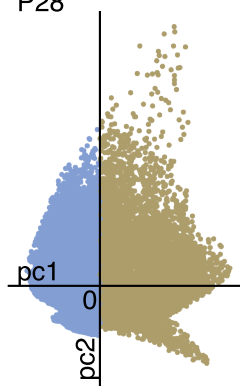

horizontal

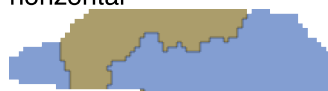

P28

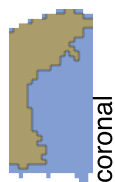

sagittal

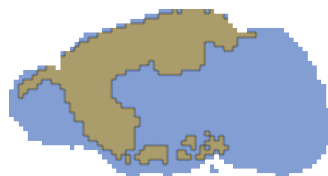

horizontal

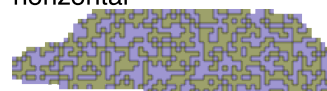

P28

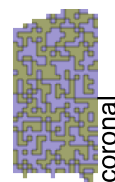

sagittal

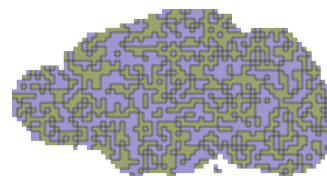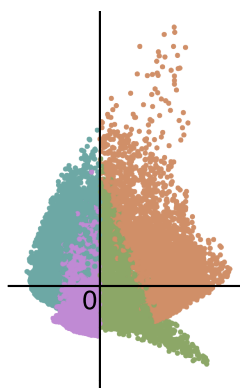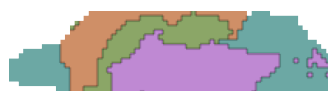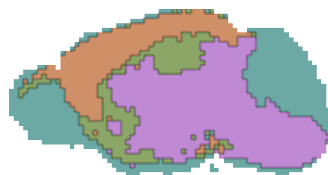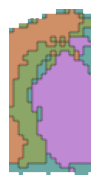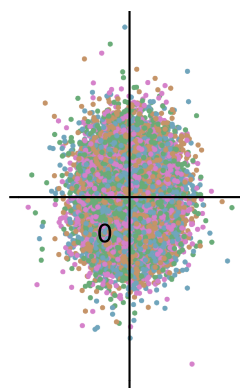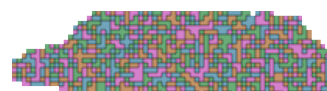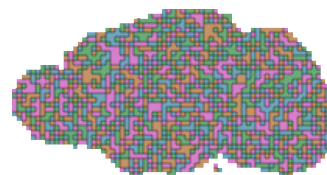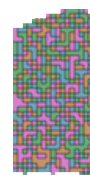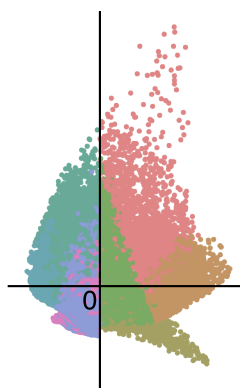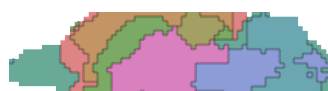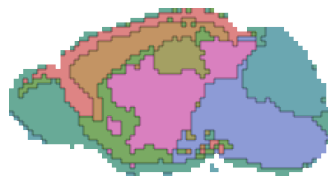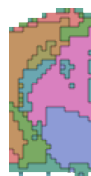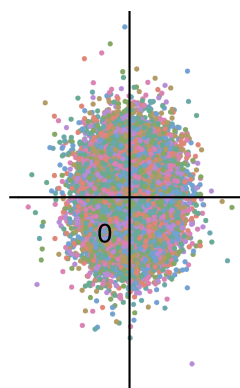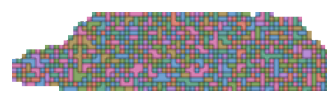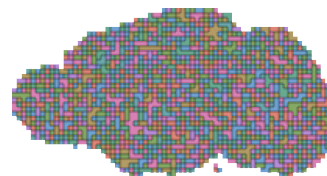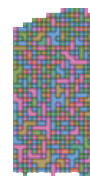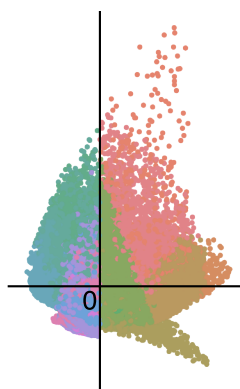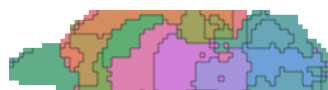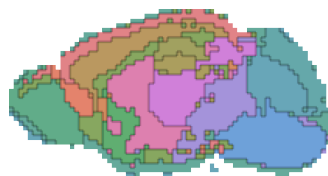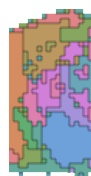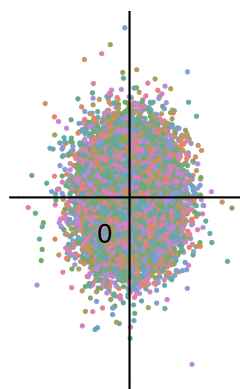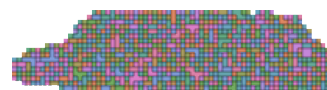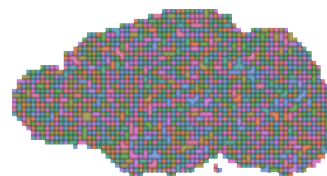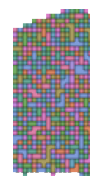

Supplement: S9 Fig — Analysis and depiction as in Fig 5. Bottom matrix shows pairwise correlation coefficient among components within the hierarchy at the displayed depths. (Similar to the bottom triangle in S2 Fig). (PDF) [file pcbi.1010382.s009.pdf]

P56

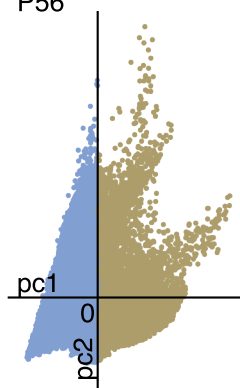

horizontal

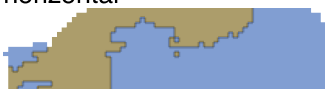

P56

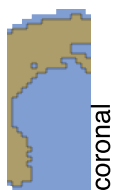

sagittal

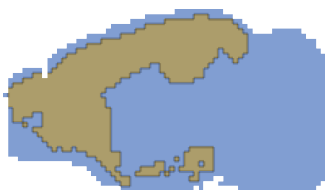

horizontal

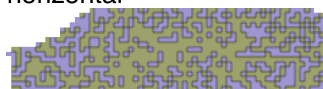

P56

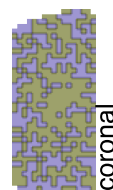

sagittal

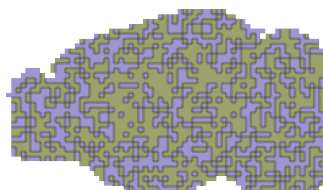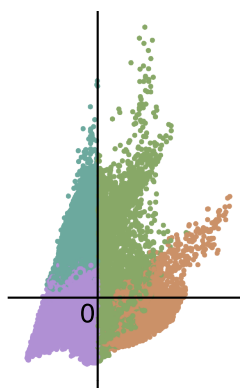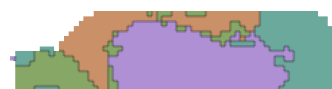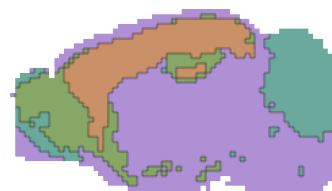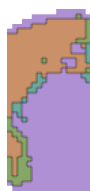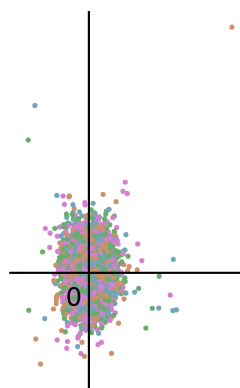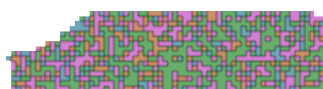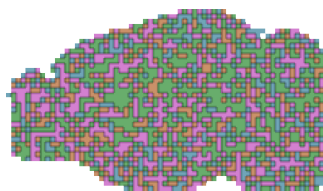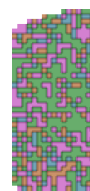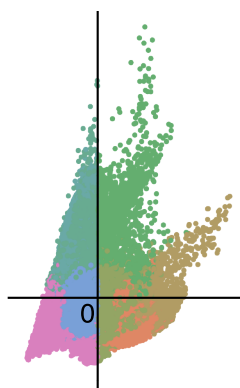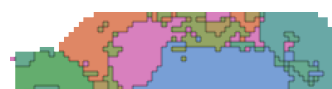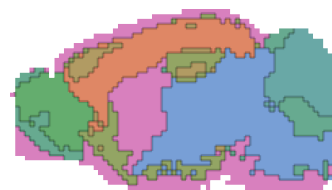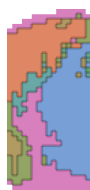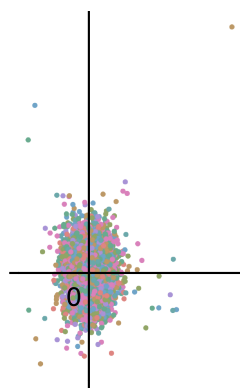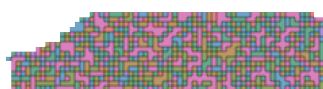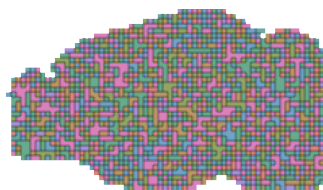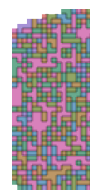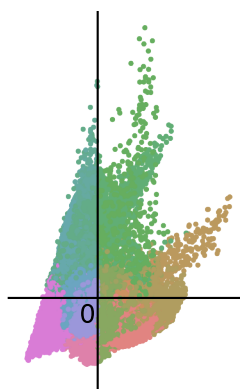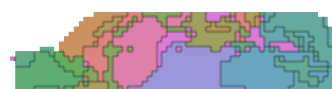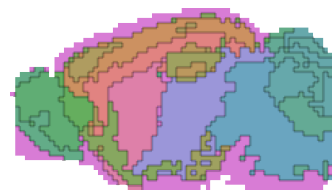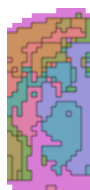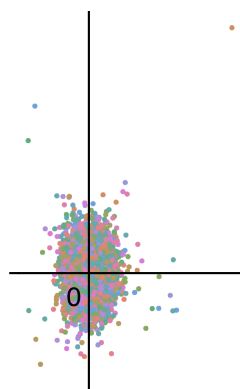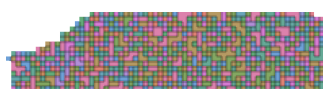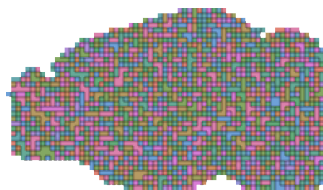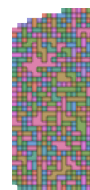

Supplement: S10 Fig — Analysis and depiction as in Fig 5. Bottom matrix shows pairwise correlation coefficient among components within the hierarchy at the displayed depths. (Similar to the bottom triangle in S2 Fig). (PDF) [file pcbi.1010382.s010.pdf]
