# Supplementary material for: Constructive connectomics: How neuronal axons get from here to there using gene-expression maps derived from their family trees
Source: PLoS Comput Biol. 2022 Aug 25;18(8):e1010382. doi: 10.1371/journal.pcbi.1010382 (PMC9409546; doi:10.1371/journal.pcbi.1010382)
Supplement: S3 Fig — Analysis and depiction as in Fig 5. Bottom matrix shows pairwise correlation coefficient among components within the hierarchy at the displayed depths. (Similar to the bottom triangle in S2 Fig). (PDF) [file pcbi.1010382.s003.pdf]

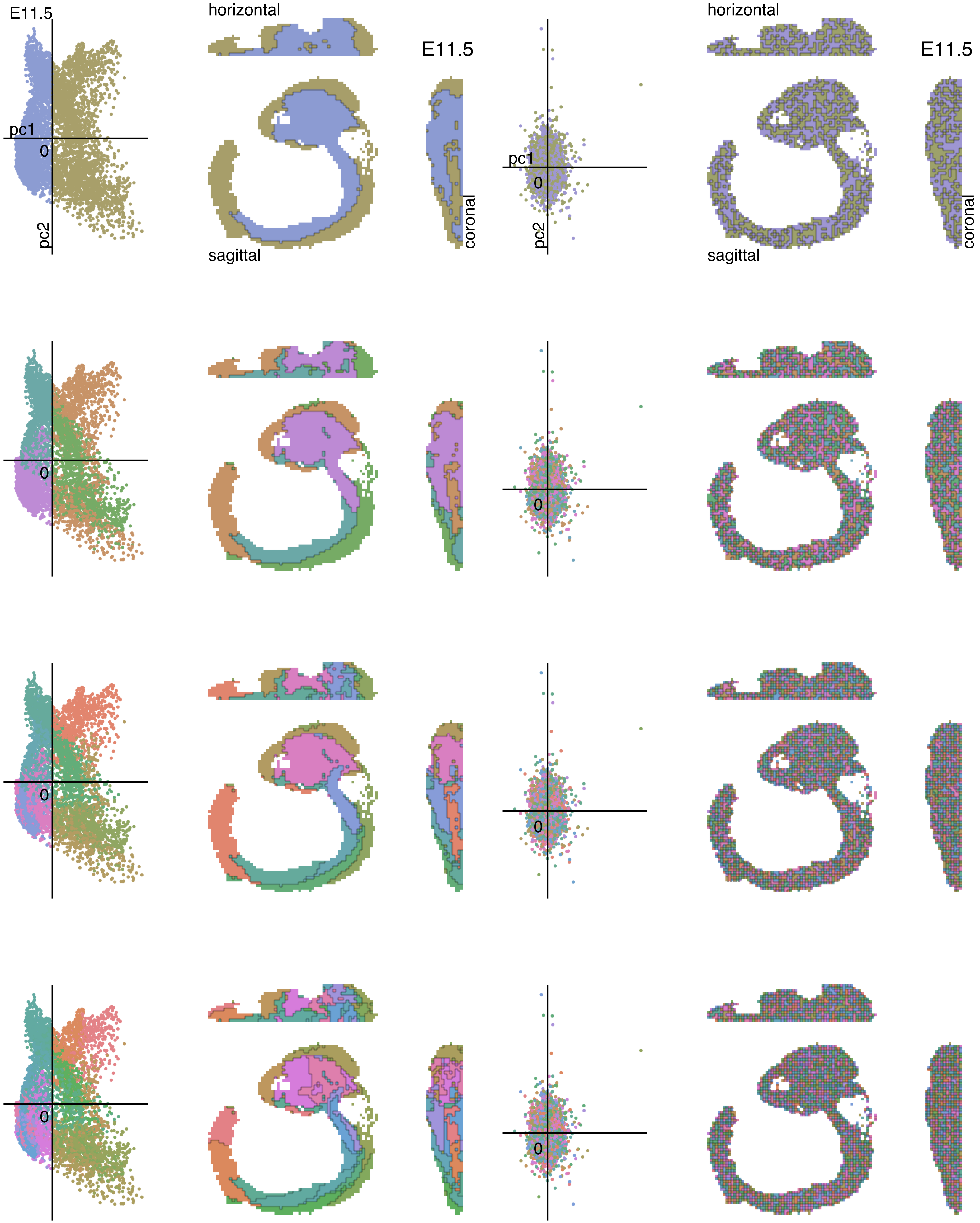

E11.5
pc1
0
pc2
horizontal
sagittal
E11.5
coronal
pc1
0
pc2
horizontal
sagittal
E11.5
coronal
0
0
0
0
0
0
